# Does Prostate Artery Embolization (PAE) Improve Voiding Symptoms, Storage Symptoms, or Both?

**DOI:** 10.1007/s00270-019-02298-3

**Published:** 2019-08-22

**Authors:** Drew Maclean, Mark Kong, Joel Lim, Sachin Modi, Mark Harris, Timothy Bryant, Nigel Hacking

**Affiliations:** 1 Department of Interventional Radiology, University Hospital Southampton, Tremona Road, Southampton, SO16 6YD UK; 2 University of Southampton Medical School, 12 University Road, Southampton, SO17 1BJ UK; 3 Department of Urology, University Hospital Southampton, Tremona Road, Southampton, SO16 6YD UK

**Keywords:** PAE, Prostate, Embolization, Storage, Voiding, Symptoms

## Abstract

**Introduction:**

Many studies have looked at global changes in the International Prostate Symptom Score (IPSS) following PAE; however, no studies have examined the breakdown between storage and voiding symptoms. We aimed to explore the extent to which PAE improves storage symptoms in relation to voiding symptoms.

**Method:**

This single-center, prospective cohort study recruited consecutive patients undergoing PAE from June 2012 to June 2016. The IPSS breakdown was recorded pre-PAE, at 3 months and 12 months post-PAE. Planned statistical analysis included the paired *t* test.

**Results:**

A total of 43 patients were recruited (mean age 64.72 ± 6.27, prostate volume 88.65 ± 37.23 cm^3^, IPSS 23.02 ± 5.84, QoL 4.98 ± 1.01, PSA 4.2 ± 2.8). Storage symptoms were more frequently the most severe symptom (58.1%). Voiding score (13.35–5.39, *p *< 0.001) and storage score (9.67–5.08, *p *< 0.001) both improved; however, voiding improved to a greater extent (1.9 vs. 1.5 mean per question, *p *= 0.023). PAE was most consistent when improving storage symptoms (‘Urgency’ improved in 86% patients, ‘Frequency’ and ‘Nocturia’ 77%).

**Conclusion:**

Storage symptoms are a significant problem for patients with benign prostatic obstruction. PAE is an effective treatment for both storage and voiding symptoms. More research is needed to evaluate how this compares with surgical techniques.

## Introduction

Prostate artery embolization (PAE) has emerged as an effective treatment for lower urinary tract symptoms (LUTS) due to benign prostatic obstruction (BPO) [1]. A multitude of studies have demonstrated a global improvement in the International Prostate Symptom Score (IPSS) following PAE [1, 2]. However, no studies have examined precisely which symptoms improve after PAE, or whether storage symptoms improve at all. Table 1 demonstrates the breakdown of storage and voiding symptoms in the IPSS questionnaire.

**Table 1:** IPSS questionnaire (each question is scored from 0 to 5 in terms of severity (0—not at all, to 5—all the time)

Symptom	Question
*Storage symptoms*	
Frequency	How often have you had to urinate less than every 2 h?
Urgency	How often have you found it difficult to postpone urination?
Nocturia	How many times did you typically get up at night to urinate?
*Voiding symptoms*	
Intermittency	How often have you found you stopped and started again several times when you urinated?
Weak stream	How often have you had a weak urinary stream?
Straining	How often have you had to strain to start urination?
Incomplete emptying	How often have you had the sensation of not emptying your bladder?

Understanding which symptoms improve following PAE is essential for counseling patients prior to the procedure and advising them which symptoms they might expect to improve. It may also enhance our understanding of the mechanism of action of PAE, or aid the design of robust selection criteria for PAE itself.

Storage symptoms are a substantial problem for patients with bladder outflow obstruction, impacting quality of life to a significantly greater extent than patients with voiding symptoms alone [3, 4]. Following transurethral prostatectomy (TURP), storage symptoms still persist in 18–51% of patients and are not improved to the same extent as voiding symptoms [5, 6]. If PAE proves a more effective treatment for storage symptoms than surgery, this could represent an indication for PAE over surgery. On the other hand, if this study identifies that certain symptoms are not treated sufficiently by PAE, patients with those symptoms would be more suited to surgery.

**Fig. 1:**
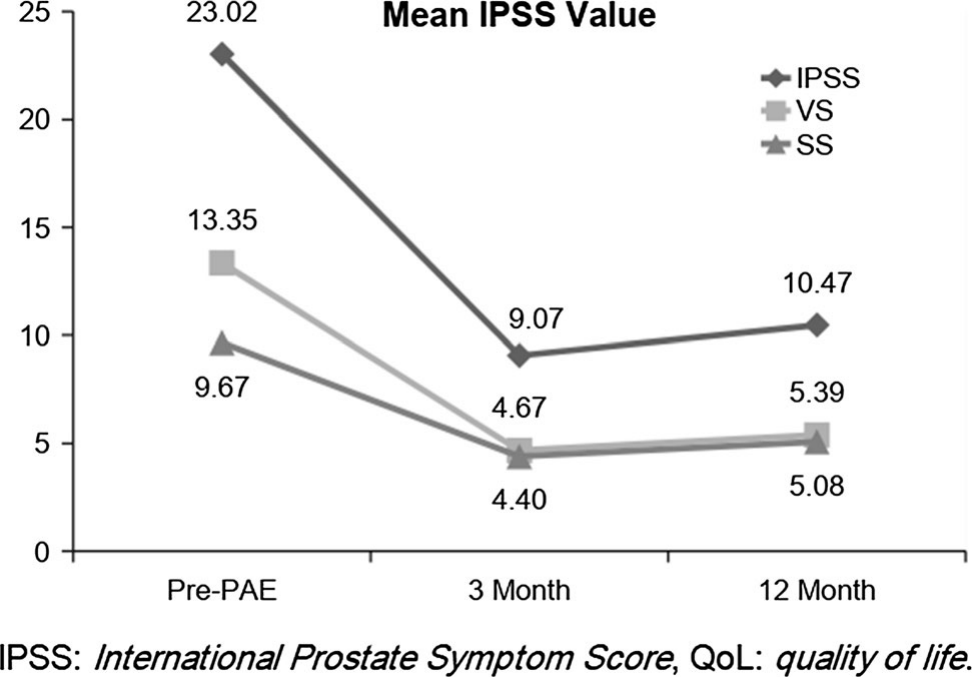
Comparison of changes in SS, VS and IPSS. *SS* storage symptoms, *VS* voiding symptoms, *IPSS* International Prostate Symptom Score at Pre-PAE baseline, 3 months post and 12 months post-PAE

We therefore aimed to identify which symptoms improve after PAE, through a prospective study focusing on the breakdown of all questions in the IPSS, with a follow-up at 3 and 12 months (Fig. 1).

## Materials and Methods

### Study Population

This single-center, prospective cohort study assessed 43 consecutive patients from June 2012 to June 2016. All patients underwent prostate artery embolization to treat lower urinary tract symptoms (LUTS) secondary to an enlarged prostate with formal urodynamic-confirmed bladder outflow obstruction (BOO). Health Research Authority approval was granted by the local research ethics committee, and adherence to the ethical principles of the Helsinki Declaration was maintained at all times.

Three consultant interventional radiologists (NH, TB, SM) performed all procedures. Patients consulted with an interventional radiologist and urologist, (in addition to a multidisciplinary team discussion) prior to the decision to treat with PAE. Inclusion criteria included patients aged 50–80, with IPSS > 14 or QoL > 3, prostate volume > 40 ml, eGFR > 45 ml min^−1^ m^−2^. Malignancy needed to be excluded first if PSA > 4 ng ml^−1^.

### Patient Evaluation

#### Baseline Assessment

All variables and data collected for the purposes of the study were recorded prospectively. In terms of symptom assessment, International Prostate Symptom Score (IPSS), quality of life-related symptoms (QoL) and International Index of Erectile Function (IIEF) were completed by patients at baseline. Uroflowmetry (peak urinary flow, *Q*_max_; post-void residual volume, PVR) and serum prostatic-specific antigen (PSA) were performed prior to PAE, in addition to prostate size estimation through pre-procedural CT.

#### Outcome Measures

All patients were followed up to 12 months post-procedure. Technical success was considered to be either unilateral or bilateral embolization of the prostatic arteries confirmed by selective angiogram. In terms of symptom assessment, IPSS questionnaires were performed at 3 and 12 months post-PAE. Categorical IPSS scores were recorded and divided into storage and voiding symptoms. Due to the presence of more voiding than storage symptoms (4 vs. 3), mean values per question were also calculated. In addition, non-formal uroflowmetry was conducted at 1, 3 and 6 months post-procedure.

#### Embolization Technique

All patients underwent CT angiogram prior to the procedure. Reference was then made to the CT images in the interventional suite during the case.

Siemens Artis Zee Ceiling angiography suite was used. Sterile technique and local anesthetic are utilized for all cases. Although exact methodology varied between practitioners and patients, several common steps were used in all cases. A 5Fr vascular sheath is inserted following unilateral (most routinely right sided) common femoral artery (CFA) access. Contralateral internal iliac artery cannulation was first attempted using a 4Fr Cobra C2 Tempo 4 catheter (Cordis, Freemont, CA) or other catheter depending on the anatomy. Prostatic artery selection and catheterization were normally achieved using a 2.5Fr Cantata (Cook, Bloomington, IN) or 2.4Fr Direxion (Boston Scientific, Marlborough, MA).

Cone-beam CT angiography was utilized to identify arterial anastomoses with adjacent organs. If potential nontarget embolization was considered a risk due to the presence of anastomoses, protective coil embolization of the particular vessel was performed when technically possible. Alternatively, a stable catheter position was achieved distal to the anastomosis prior to embolization. Embolization of the prostate was then carried out with non-spherical PVA (polyvinyl alcohol) particles (100 and/or 200 microns, Cook Medical, Bloomington, IN) or alternatively spherical Embozenes 400 micron or smaller (Boston Scientific, Marlborough, MA).

### Statistical Analysis

Categorical IPSS scores were recorded and evaluated with the paired *t* test. Pearson’s bivariate correlation was used to assess for correlation of variables. For differences between independent variables, the independent means *t* test was utilized. Statistical analysis was performed using SPSS 16.0 (SPSS Inc, Chicago, IL, USA). All variables were assessed for normality using the Shapiro–Wilk test. A *p* value < 0.05 was taken to be significant.

## Results

### Baseline Characteristics

A total of 43 consecutive patients were identified (Table 2) (mean age 64.72 ± 6.27, prostate volume 88.65 ± 37.23 cm^3^, IPSS 23.02 ± 5.84, QoL 4.98 ± 1.01, PSA 4.2 ± 2.8). Voiding symptoms contributed a mean 13.35 points to the overall initial IPSS of 23.02, whereas storage symptoms contributed a mean 9.67 points. Voiding symptoms were scored at 3.4 out of 5 per IPSS question, and storage symptoms were scored at 3.2 out of 5 per question.

**Table 2:** Baseline clinical characteristics of participants prior to PAE

Characteristics	Participant results Mean ± SD (range)
Age at procedure (yr)	64.72 ± 6.27
Prostate volume (ml)	88.65 ± 37.23
PSA (ng ml^−1^)	4.2 ± 2.8
IPSS	23.02 ± 5.84
VS	13.35 ± 4.15
SS	9.67 ± 2.89
QoL	4.98 ± 1.01

*PSA* serum prostate-specific antigen, *IPSS* International Prostate Symptom Score, *VS* voiding symptoms, *SS* storage symptoms, *QoL* quality of life

### Symptom Improvement

Technical success was achieved in all patients, and they were all followed up to 12 months post-PAE. At 3 months, a mean reduction of 12.12 ± 7.17 in total IPSS was demonstrated from 23.02 ± 5.84 to 9.07 ± 5.88. At 12 months post-PAE, overall IPSS score reduced to 10.47 ± 6.40 (pre-PAE 23.02 ± 5.84). The quality of life score also reduced significantly from 4.98 ± 1.01 to 1.91 ± 1.36.

### Storage Symptoms and Voiding Symptoms

Statistically significant reductions were seen in both voiding symptoms and storage symptoms. Mean voiding IPSS reduced from 13.35 to 5.39 (paired *t* test *p *< 0.001), and mean storage IPSS reduced from 9.67 to 5.08 (paired *t* test *p *< 0.001). When comparing the mean reduction per question between voiding and storage symptoms, a statistically greater reduction was seen in voiding symptoms (1.9 vs. 1.5, *p *= 0.023). However, 25/43 (58.1%) patients’ most severe symptom (highest scoring symptom on the questionnaire) prior to PAE was a storage symptom.

### Individual Symptoms

The individual symptoms which demonstrated the greatest reduction at 12 months post-PAE were both voiding symptoms (Table 3). ‘Incomplete emptying’ reduced from mean 3.67 to 1.39 (*p *< 0.001), a reduction of 2.28. ‘Intermittency’ reduced a similarly significant amount, from 3.47 to 1.26, a reduction of 2.21 (*p *< 0.001).

The symptoms that most frequently saw a decrease (≥ 1 IPSS point) were all storage symptoms: Urgency (86% of patients saw an improvement in this symptom score), Frequency (77%) and Nocturia (77%).

**Table 3:** Mean values of IPSS parameters pre- and post-PAE

Symptoms	Mean Score		
	Pre-PAE	3 months post	12 months post
Incomplete emptying	3.67 ± 1.38	1.16 ± 1.17	1.39 ± 1.50
Frequency	3.40 ± 1.38	1.51 ± 1.08	1.89 ± 1.31
Intermittency	3.47 ± 1.42	1.30 ± 1.42	1.26 ± 1.45
Urgency	3.23 ± 1.51	1.19 ± 1.14	1.29 ± 1.29
Weak stream	4.10 ± 1.13	1.70 ± 1.46	2.13 ± 1.71
Straining	2.12 ± 1.43	0.51 ± 0.91	0.61 ± 1.05
Nocturia	3.05 ± 1.29	1.70 ± 1.10	1.89 ± 1.11
IPSS	23.02 ± 5.84	9.07 ± 5.88	10.47 ± 6.40
QoL	4.98 ± 1.01	1.91 ± 1.36	2.08 ± 1.48

### Baseline Symptoms and Correlation with Overall IPSS Reduction

The pre-PAE individual symptom scores which correlated best with overall IPSS reduction were storage symptoms. These symptoms were Frequency (Pearson’s bivariate correlation *r *= 0.44, *p *= 0.006) and Urgency (*r *= 0.54, *p *= 0.001).

## Discussion

By observing only the total IPSS change after prostate intervention, studies are perhaps ignoring important trends in the IPSS subscores which could highlight important differences in symptomatic improvement between BPH therapies. This in turn could help patients decide which treatment has the best chance of relieving their most bothersome symptom (Table 3).

### Storage Symptoms

Storage symptoms are a considerable issue for PAE patients with the majority of patients (58.1%) having a storage symptom as their most severe complaint. Fortunately, patients with severe Frequency and Urgency (both storage symptoms) are most likely to have a greater overall benefit (global IPSS reduction) from PAE. Furthermore, PAE was demonstrated to significantly reduce all storage symptoms at 3 and 12 months and was in fact most consistent in improving all 3 storage symptoms rather than voiding symptoms (Urgency improved in 86% patients, Frequency and Nocturia 77%).

### Voiding Symptoms

Admittedly, the reduction in storage symptoms is not as great as the reduction in voiding symptoms (mean 1.9 vs. 1.5 reduction per symptom, *p *= 0.023). This is perhaps to be expected as an improvement in flow (and therefore flow-related symptoms) is the main benefit to be gained from PAE. In this regard, PAE has similar response proportions (storage/voiding ratio) as TURP [4, 5].

### Individual Symptoms

A significant improvement was shown in each individual symptom category, even though voiding symptoms improved the most. Therefore, patients with particularly troublesome individual symptom can be counseled that PAE is highly likely to treat their particular symptom, regardless of whether it is a voiding or a storage symptom. Despite this, the voiding symptoms of ‘incomplete emptying’ and ‘intermittency’ demonstrated the greatest reductions in severity, and patients with these symptoms can therefore expect a considerable improvement.

### Study Limitations

As no direct comparison with TURP has been made, this study has not shown that PAE has superiority for patients with storage symptoms over TURP. We have shown that the proportional improvement in storage/voiding symptom improvement is similar to previous studies on TURP; however, a direct comparison cannot be made without a purpose-built study. Future comparative trials should therefore aim to collect data on the IPSS breakdown and how it compares with surgical therapies.

Finally, these patients were only been followed up to 12 months, and therefore, a longer-term assessment of voiding/storage symptoms cannot be made. However, long-term data suggest that outcomes are similar for the past 12 months [7]. Changes in individual symptoms are therefore also unlikely to alter in magnitude.

### Other Implications

Storage symptoms often have a more complex and multifactorial causation when compared with voiding symptoms [8], and BPO occasionally is not the cause of storage symptoms. Radiologists therefore need to be aware that patients with only storage symptoms (rather than voiding and storage symptoms combined) may be due to other etiologies. Formal urodynamics (with invasive bladder pressure monitoring) remain the gold-standard method of demonstrating bladder outflow obstruction. This investigation is therefore perhaps more relevant in patients with significant storage symptoms and minimal voiding symptoms, as other underlying etiologies can be the cause and need to be excluded [8].

## Conclusion

PAE improves all urinary symptoms addressed on the IPSS questionnaire, and patients can be reassured that all urinary symptoms are likely to improve post-PAE. We have also shown that storage symptoms are a considerable issue for patients prior to PAE, with most patients having a storage symptom which is their most severe complaint. Reassuringly, PAE consistently reduces these storage symptoms, although the magnitude of benefit is greater in voiding symptoms, similar to TURP.
